# A forest-based feature screening approach for large-scale genome data with complex structures

**DOI:** 10.1186/s12863-015-0294-9

**Published:** 2015-12-23

**Authors:** Gang Wang, Guifang Fu, Christopher Corcoran

**Affiliations:** Department of Mathematics and Statistics, Utah State University, 3900 Old Main, Logan, 84322 UT USA

**Keywords:** Feature screening, GWAS, Epistasis, Random forest, Large-scale modeling

## Abstract

**Background:**

Genome-wide association studies (GWAS) interrogate large-scale whole genome to characterize the complex genetic architecture for biomedical traits. When the number of SNPs dramatically increases to half million but the sample size is still limited to thousands, the traditional *p*-value based statistical approaches suffer from unprecedented limitations. Feature screening has proved to be an effective and powerful approach to handle ultrahigh dimensional data statistically, yet it has not received much attention in GWAS. Feature screening reduces the feature space from millions to hundreds by removing non-informative noise. However, the univariate measures used to rank features are mainly based on individual effect without considering the mutual interactions with other features. In this article, we explore the performance of a random forest (RF) based feature screening procedure to emphasize the SNPs that have complex effects for a continuous phenotype.

**Results:**

Both simulation and real data analysis are conducted to examine the power of the forest-based feature screening. We compare it with five other popular feature screening approaches via simulation and conclude that RF can serve as a decent feature screening tool to accommodate complex genetic effects such as nonlinear, interactive, correlative, and joint effects. Unlike the traditional *p*-value based Manhattan plot, we use the Permutation Variable Importance Measure (PVIM) to display the relative significance and believe that it will provide as much useful information as the traditional plot.

**Conclusion:**

Most complex traits are found to be regulated by epistatic and polygenic variants. The forest-based feature screening is proven to be an efficient, easily implemented, and accurate approach to cope whole genome data with complex structures. Our explorations should add to a growing body of enlargement of feature screening better serving the demands of contemporary genome data.

## Background

High-throughput genotyping techniques and large data repository capability give genome-wide association studies (GWAS) great power to unravel the genetic etiology of complex traits. With the number of Single Nucleotide Polymorphisms (SNPs) per DNA array growing from 10,000 to 1 million [1], ultra-high dimensionality is one of the grand challenges in GWAS. The prevailing strategies of GWAS focus on single-locus model [2, 3]. However, most complex traits are regulated by polygenetic variants, which decreases the power of most popular traditional *p*-value based approaches [4–7].

Epistasis [2, 8, 9], defined as the interactive effects of two or more genetic variants (i.e. the effect of one genetic variant is suppressed or enhanced by other genetic variants), has received growing attention in GWAS due to increasing evidence of its important role in the development of complex diseases [7, 10–12]. Epistasis will likely bring key breakthroughs for detecting more susceptible loci for various real life scenarios and for explaining larger heritability of traits [13–16]. Many approaches have already been developed for detecting epistasis [17–20]. Despite the fact that these approaches work nicely for detecting epistasis with a moderate number of SNPs (*n*>*p*), they quickly lose power and suffer from computational burden when the dimension is ultrahigh (*n*>>*p*) [12].

There exists a big gap between current statistical modeling of big data and the real demand of contemporary entire genome data. Fan et al. elaborately introduced the unusually big challenges in computational cost, statistical estimation accuracy, and algorithm stability caused by ultrahigh dimensional data [21–23]. The population covariance matrix may become ill conditioned as dimension grows as multicollinearity grows with dimensionality. As a result, the number and extent of spurious correlations between a feature and response increase rapidly with increasing dimension because unimportant features are often highly correlated with a truly important one. What increases the difficulty is that multiple genetic variants affect the phenotype in an interactive or correlative manner but each have a weak marginal signal. Additionally, without any priori information, modeling and searching all possible pairwise and higher order interactions is intractable when the number of features is very large. For example, there will be around 8 million pairs involved when simply considering 2-way interactions for only 4000 SNPs [24].

Feature Screening brings about a revolutionary time in statistics due to its advantages in handling ultrahigh dimensional data. It also fills the gap between traditional statistical approaches and demands of contemporary genomics [25]. The sparsity principle (only a small number of SNPs associate with the phenotype) of the whole genome data matches well with the goal of the feature screening. It has been confirmed that the computational speed and estimation accuracy are both improved after dimension is reduced from ultrahigh to moderate size [26]. The computational burden reduces dramatically, from a huge scale (say exp{*O*(*n*^*h*^)}) to *o*(*n*). Most important of all, aforementioned traditional statistical approaches regain their power and feasibility after feature screening removes the majority of confounding noises. Fan and Lv proposed sure independence screening (SIS) and iterated sure independence screening (ISIS) [26] to overcome the challenges of ultra-high dimension. SIS is shown to have the sure screening property (all truly important predictors can be selected with the probability tending to one as the sample size asymptotically diverges to *∞* [26, 27]) for the case of *n*>>*p*. Fan and Song developed SIS for generalized linear models [28]. Li et al. proposed distance correlation learning (DC-SIS) without assuming linear relation or restricting data type [27, 29]. Liu et al. proposed conditional correlation sure independence screening (CC-SIS) to adjust the confounding effect of a covariate [30].

Although the advantages of the feature screening have been sufficiently shown, almost all current feature screening approaches assign univariate rankings to consider the individual effect of each feature and hence neglect features that have weak marginal but strong joint or interactive effects. In addition, most existing feature screening approaches are not well-designed for examining two, three, or higher-order interactive structures and nonlinear structures. As an alternative direction, Random Forest (RF) overcomes the aforementioned drawbacks of feature screening. RF uncovers interactive effects even if the relevant features only have weak marginal signals [31]. Each hierarchical decision tree within the RF explicitly represents the attribute interaction of features through the branches of the tree. As a result, as more and higher order interactive SNPs are added to the model, the superiority of RF increases. In particular, RF was claimed to outperform Fisher’s exact test when interactive effects exist [32]. RF can be flexibly modeled to both continuous and categorical phenotype and nonlinear structures without assuming any model structure or interaction forms.

The aim of this article is to assess the performance of a forest-based feature screening approach for large-scale whole genome data with complex genetic structures such as epistastic, polygenic, correlative, and nonlinear effects. The key problem that we emphasize is to select a manageable number of important candidates from an ultrahigh dimension of SNP pool, while keeping the case of strong marginal signal, the case of of weak marginal but strong interactive or correlative SNPs, and keeping both linear and nonlinear structures. Unlike the traditional *p*-value based Manhattan plot, we view the significance of SNPs using permutation variable importance measure (PVIM). The PVIM based Manhattan plot can provide as much helpful information as the traditional *p*-value based Manhattan plot, additionally it considers the individual effect of each SNP as well as accounting for the mutual joint effects of all other SNPs in a multivariate sense. In current literature, a few studies have already assessed the performance of RF for detecting epistasis [32–35], but they all focused on binary/case-control phenotype. Additionally, current literature simply consideres two-way interaction simulations and it is not clear whether or not RF can perform well for more complex interactions. Instead, we explored the performance of RF for quantitative/continuous traits and additionally increased the complexity level by considering nonlinearity, correlation, and more difficult interaction simultaneously.

## Results and discussion

### Power simulation

To illustrate the power of RF as a feature screening tool for detecting correlative, nonlinear, and interactive effects, we designed four different simulation settings to control linear vs nonlinear, constant vs functional, and additive vs interactive features. We compare RF with five popular feature screening tools, SIS [26], ISIS [26], CC-SIS [30], ICC-SIS [30], and DC-SIS [27]. In order to make the comparisons fair, we keep some of their original simulation settings the same, as well as design other settings different to accommodate the emphasis of this study.

The sample size *n* is set to be 200. Let ***X***=(*x*_1_,…,*x*_*p*_)^*T*^∼*N*(**0**,*Σ*) be the feature matrix with dimension *p*=1000. By controlling the component *σ*_*ij*_=*ρ*^|*i*−*j*|^,*i*,*j*=1,…,*p* of covariance matrix *Σ*, the correlations among features are introduced. All the values of *β*s are zero, except the truly causative features. Among the 1000 features, we set the first five to be truly associated with phenotype and all others be noise by letting

(1) $$ Y= \beta_{1}x_{1}+\beta_{2}x_{2}+\beta_{3}x_{3}+\beta_{4}x_{4}x_{5}+\epsilon,   $$

for the linear and moderate interactive setting, and

(2) $$ Y = \beta_{1}{x_{1}^{2}}+\beta_{2}x_{2}x_{3}+\beta_{3}x_{4}x_{5}+\epsilon.   $$

for the nonlinear and strong interactive setting. The noise *ε* is randomly generated from white noise *N*(0,1).

- Simulation 1

- For Sim 1, we consider three linear and one interactive terms with constant parameters. i.e. *Y* is generated based on Eq. (1), *ρ*=0.4, and *β*s are set to be *β*=(0.5, 0.8, 1, 2).

- Simulation 2

- For Sim 2, we consider one nonlinear and two interactive terms with constant parameters. i.e. *Y* is generated based on Eq. (2), *ρ*=0.4, and *β*s are set to be *β*=(2, 3, 4).

- Simulation 3

- For Sim 3, we consider three linear and one interactive terms with functional parameters. i.e. *Y* is generated based on Eq. (1), *ρ*=0.4, and *β*s are generated by *β*_1_=2+(*u*+1)^3^, $\beta _{2} = \frac {2u^{2}+3}{2}$, $\beta _{3} = e^{\frac {4u}{u+4}}$, and $\beta _{4} = \cos \left (\frac {8u^{2}}{2}\right)+2$. In order to introduce the correlation between each feature and a covariate *u*, we generate (*u*^∗^,*X*)∼*N*(**0**,*Σ*^∗^), here *Σ*^∗^ is (*p*+1)×(*p*+1) dimension using similar AR(1) structure as above *Σ*. Then we generate *u* by *u*=*Φ*(*u*^∗^), here *Φ*(.) is the cumulative distribution function (cdf) of the standard normal distribution. By the theoretical properties of cdf, *u* follows a uniform distribution *U*(0,1) and is correlated with *X*. The functional parameter *β*(*u*) is useful to explain personalized covariate effects that vary for different individuals due to different genetic information and other factors [30].

- Simulation 4

- For Sim 4, we consider one nonlinear and two interactive terms with functional parameters. i.e. *Y* is generated based on Eq. (2), *ρ*=0.4, and *β*s are generated by $\beta _{1} = 2+\cos \left (\frac {\pi (6u-5)}{3}\right)$, $\beta _{2} = (4-4u)e^{\frac {3u^{2}}{3u^{2}+1}}$, and *β*_3_=*u*+2. *u* and *X* are generated using the same rule as Sim 3. This setting has the hardest conditions that hinder most approaches from detecting the truly causative features.

The comparisons were assessed based on 100 simulation replications. Three traditional criteria that frequently appeared in feature screening literature [27], *R*, *p*, and *M*, are used to compare the performances of six approaches.

- *R*_*j*_, *j*=1,…,5, is defined as the average rank of each causative feature *x*_*j*_ for 100 replications. Since the most important feature is ranked as top one, smaller *R* for causative features means better performance.
- *M*= max *R*_*j*_, *j*=1,…,5, is defined as the minimum size of the candidate containing all five causative features. Therefore, *M* close to five means good performance. Like other feature screening studies, we also compared the 5, 25, 50, 75, and 95 *%* quantiles of *M* for the 100 replications. These quantiles display how effective each approach is during selection process.
- *d* is defined as the pre-specified number of candidates that will be chosen as important. In real life data, we do not know the minimum size containing all causative features. Liu et al. [30] suggested to use the multiplier of the integer part of *d*=[*n*^4/5^/*log*(*n*^4/5^)]. i.e. for *n*=200, *d* is suggested to be 16, 32, and 48, and so on. We use the same values to make the comparisons fair.
- *p*_*j*_, *j*=1,…,5, is defined as the percentage of each *x*_*j*_ being successfully selected within size *d* among 100 replications. The larger *p*_*j*_, the more accurate (higher individual power).
- *p*_*a*_ is defined as the percentage of all five causative features being successfully selected within size *d* among 100 replications. The larger *p*_*a*_, the more accurate (higher overall power).

The comparative results of the constant parameters for Sim 1 and Sim 2 are summarized in Tables 1, 2 and 3. Table 1 reports the average rank of all five causative features. For Sim 1, the first three features have linear marginal effects but *x*_4_ and *x*_5_ have interactive effects. The marginal effect of *x*_1_ is designed to be smaller than that of *x*_2_ or *x*_3_ by setting *β*_1_=0.5,*β*_2_=0.8, and *β*_3_=1. For the simplest scenario (strong linear marginal effects of *x*_2_ and *x*_3_), all six approaches achieve remarkable results with the average ranks *R*_2_ and *R*_3_ all less than 2. It means that all six feature screening approaches successfully locate these two causative features as the top two. For the weak linear marginal effect of *x*_1_, it seems that the iterative approaches perform worse than their corresponding original approaches, say ISIS 39.29 versus SIS 12.21 and ICC-SIS 43.75 versus CC-SIS 12.81. In the reports of Fan et al. and Liu et al., the iterative procedure greatly improved the results compared to that of previous iterative procedures under all their reported scenarios [26, 30]. Therefore, we still agree with the advantages of iterative approaches, but maintain that our new findings can help readers gain insight about the pitfalls and benefits of each approach. The six approaches behave dramatically different for the interactive terms *x*_4_ and *x*_5_. Both *R*_4_ and *R*_5_ obtained from the first four approaches are very large, which means that they rank hundreds of other candidates before these two causative features. Compared to the 412.43 of ISIS and 179.77 of CC-SIS, RF achieves a rank as small as 4.06. Observing the last row of Table 1, we conclude that RF detects all five causative features using the smallest number of candidates (less than 9 in average). One more thing worth mentioning is that RF ranks the features with strong interactive but weak marginal effects (3.72 for *x*_4_ and 4.06 for *x*_5_) more important than features with weak marginal effects (8.65 for *x*_1_). The overall importance rank of RF combines all related effects rather than simply considering marginal importance.

**Table 1:** The average rank of each causative feature, *R*_*j*_, for Simulation 1 & 2

			Sim1					Sim2		
METHOD	R1	R2	R3	R4	R5	R1	R2	R3	R4	R5
SIS	12.21	1.56	1.51	143.14	322.16	359.17	360.41	398.89	340.45	428.30
ISIS	39.29	1.56	1.51	250.98	412.43	432.97	456.97	481.98	426.94	502.13
CC-SIS	12.81	1.59	1.48	60.31	179.77	168.57	242.27	242.85	258.39	369.68
ICC-SIS	43.75	1.59	1.48	129.80	259.34	237.70	362.12	382.58	368.86	400.27
DC-SIS	5.95	1.59	1.48	7.93	19.58	3.51	21.07	32.86	7.44	14.79
RF	8.63	1.91	1.67	3.72	4.06	2.80	8.59	10.70	4.66	7.85

**Table 2:** The quantiles of *M*, for Simulation 1 & 2

			Sim1					Sim2		
METHOD	5 %	25 %	50 %	75 %	95 %	5 %	25 %	50 %	75 %	95 %
SIS	15.60	72.25	339.50	646.00	887.75	257.25	681.00	817.50	888.00	970.20
ISIS	14.65	331.75	597.50	756.25	958.00	555.85	766.75	875.00	954.75	986.15
CC-SIS	7.90	34.75	107.00	288.50	703.80	131.85	357.25	605.50	812.75	957.30
ICC-SIS	7.90	150.50	357.50	530.25	838.60	387.35	614.50	784.00	865.75	951.25
DC-SIS	5.00	6.00	8.00	16.25	55.20	7.00	16.50	31.00	66.50	152.60
RF	5.00	5.00	5.00	8.00	17.05	5.00	7.75	11.00	22.00	67.15

**Table 3:** The overall and individual power, *p*_*a*_ and *p*_*j*_, for Simulation 1 & 2

				Sim1						Sim2			
d	METHOD	*p*_1_	*p*_2_	*p*_3_	*p*_4_	*p*_5_	*p*_*a*_	*p*_1_	*p*_2_	*p*_3_	*p*_4_	*p*_5_	*p*_*a*_
	SIS	0.97	0.97	0.97	0.48	0.09	0.08	0.09	0.04	0.01	0.01	0.01	0.00
	ISIS	0.95	0.95	0.95	0.41	0.11	0.06	0.09	0.02	0.01	0.03	0.01	0.00
	CC-SIS	0.95	0.95	0.95	0.61	0.17	0.15	0.34	0.13	0.09	0.07	0.01	0.00
16	ICC-SIS	0.91	0.91	0.91	0.52	0.16	0.11	0.32	0.09	0.09	0.04	0.01	0.00
	DC-SIS	0.99	0.99	0.99	0.92	0.79	0.77	0.95	0.67	0.56	0.81	0.72	0.30
	RF	0.93	0.93	0.93	0.93	0.93	0.93	0.99	0.88	0.84	0.95	0.89	0.67
	SIS	0.97	0.97	0.97	0.55	0.18	0.14	0.09	0.05	0.04	0.03	0.02	0.00
	ISIS	0.95	0.95	0.95	0.42	0.12	0.07	0.09	0.02	0.01	0.03	0.01	0.00
	CC-SIS	0.95	0.95	0.95	0.67	0.29	0.22	0.34	0.17	0.15	0.08	0.04	0.00
32	ICC-SIS	0.91	0.91	0.91	0.55	0.22	0.14	0.32	0.09	0.10	0.05	0.01	0.00
	DC-SIS	0.99	0.99	0.99	0.94	0.90	0.86	0.95	0.78	0.67	0.91	0.84	0.48
	RF	0.93	0.93	0.93	0.93	0.93	0.93	0.99	0.94	0.92	0.97	0.95	0.82
	SIS	0.97	0.97	0.97	0.57	0.20	0.16	0.09	0.05	0.04	0.03	0.02	0.00
	ISIS	0.95	0.95	0.95	0.42	0.13	0.07	0.09	0.02	0.02	0.03	0.01	0.00
	CC-SIS	0.95	0.95	0.95	0.74	0.35	0.30	0.34	0.19	0.17	0.08	0.05	0.00
48	ICC-SIS	0.91	0.91	0.91	0.60	0.24	0.16	0.32	0.10	0.11	0.07	0.03	0.00
	DC-SIS	0.99	0.99	0.99	0.97	0.96	0.94	0.95	0.85	0.75	0.94	0.92	0.64
	RF	0.93	0.93	0.93	0.93	0.93	0.93	0.99	0.96	0.95	0.99	0.95	0.88

For Sim 2, *x*_1_ has a nonlinear effect and all other four features have interactive effects. This setting is much more difficult than Sim 1. As a result, all five ranks achieved by the first four approaches dramatically increased from decades in Sim 1 to hundreds in Sim 2. RF consistently performs best for this harder condition by locating all five causative features with complex structures within 11 candidates on average. Compared the results of Sim 1 and Sim 2 in Table 1, all six approaches get worse in harder conditions, but the differences of RF is negligible, with 8.63 versus 10.70. It indicates that RF is more robust than the other five approaches under harder conditions.

Table 2 reports five quantiles of *M*, the minimum size of candidates containing all the five truly causative features, among 100 simulation replicates. The first four approaches have a 95 *%* quantile as large as 958 for Sim 1 and 986 for Sim 2, meaning the detection of interactive terms fails. Among the 100 simulation replicates, the five quantiles of RF are relatively unchanged. To be more specific, 50 *%* of the replicates locate all five truly causative features using 5 candidates (a perfect match), 75 *%* of the replicates locate all five truly causative features by 8 candidates, and 95 *%* of the replicates locate truth by 17 candidates. Comparing the span from 5–95 % of these six approaches, we conclude that RF is very effective and accurate in locating important causative features.

Table 3 reports the powers achieved by three different pre-specified sizes *d*=16,32 and 48. For a small size *d*=16, RF already achieves a power as large as 93 *%*, while the first four approaches only a power of 15 *%*. When *d* triples, the power of DC-SIS increases from 77– 94 *%* but the power of RF keeps all the same as 93 *%*. Additionally, the five individual powers of RF do not differ much like other approaches. These findings confirm that RF detects all true causative features with high efficiency and high accuracy for complex structures.

The comparative results of the functional parameters for Sim 3 and Sim 4 are summarized in Tables 4, 5 and 6. Closely inspecting the results of Tables 4, 5 and 6, we find that the superiorities of RF over all other five approaches are similar as summarized in Tables 1, 2 and 3. For Sim 3, the first three features have linear marginal effects but *x*_4_ and *x*_5_ have interactive effect. The parameter *β*s are designed to be nonlinear and complex functions of a covariate *u*. For Sim 4, *x*_1_ is in nonlinear form, and the interactions are very strong because *x*_2_ interacts with *x*_3_ and *x*_4_ interacts with *x*_5_. The *β*s are designed to be more complex functions of *u*. The six approaches all do well for *x*_1_ through *x*_3_ under Sim 3, but RF beats all other five approaches under the remaining scenarios (see Tables 4, 5 and 6). DC-SIS has performed as better as RF in the first two simulations but lost its power for Sim 3 and Sim 4.

**Table 4:** The average rank of each causative feature, *R*_*j*_, for Simulation 3 & 4

			Sim3					Sim4		
METHOD	*R*_1_	*R*_2_	*R*_3_	*R*_4_	*R*_5_	*R*_1_	*R*_2_	*R*_3_	*R*_4_	*R*_5_
SIS	1.00	2.00	3.00	160.15	379.58	262.87	369.88	392.07	363.33	494.10
ISIS	1.00	2.00	3.00	353.95	518.05	311.39	416.71	485.63	428.34	461.79
CC-SIS	1.00	2.00	3.00	140.46	376.82	26.58	155.47	199.26	269.65	409.99
ICC-SIS	1.00	2.00	3.00	285.10	429.93	44.73	305.71	316.91	344.90	417.34
DC-SIS	1.00	2.01	2.99	111.32	228.75	1.35	16.93	27.88	30.67	57.52
RF	1.00	2.01	3.14	59.87	107.06	1.25	6.58	13.66	14.98	26.18

**Table 5:** The quantiles of *M*, for Simulation 3 & 4

			Sim3					Sim4		
METHOD	5 %	25 %	50 %	75 %	95 %	5 %	25 %	50 %	75 %	95 %
SIS	24.95	176.00	380.50	711.25	960.75	384.20	599.75	787.00	926.75	992.05
ISIS	225.30	425.00	624.00	827.00	956.05	361.70	688.25	796.50	917.00	983.05
CC-SIS	31.90	165.50	393.00	662.75	883.60	43.70	330.00	623.00	811.50	959.55
ICC-SIS	95.45	321.00	538.00	754.50	936.90	209.75	479.00	721.00	867.25	961.35
DC-SIS	15.00	57.50	205.50	445.25	692.85	10.00	29.00	61.50	115.00	228.30
RF	7.00	14.50	65.00	189.25	603.05	6.00	12.00	19.50	42.75	149.75

**Table 6:** The overall and individual power, *p*_*a*_ and *p*_*j*_, for Simulation 3 & 4

				Sim3						Sim4			
d	METHOD	*p*_1_	*p*_2_	*p*_3_	*p*_4_	*p*_5_	*p*_*a*_	*p*_1_	*p*_2_	*p*_3_	*p*_4_	*p*_5_	*p*_*a*_
	SIS	1.00	1.00	1.00	0.41	0.03	0.02	0.22	0.08	0.02	0.03	0.03	0.00
	ISIS	1.00	1.00	1.00	0.31	0.01	0.00	0.16	0.06	0.03	0.03	0.02	0.00
	CC-SIS	1.00	1.00	1.00	0.37	0.04	0.03	0.86	0.30	0.22	0.06	0.07	0.00
16	ICC-SIS	1.00	1.00	1.00	0.27	0.02	0.02	0.83	0.24	0.16	0.06	0.05	0.00
	DC-SIS	1.00	1.00	1.00	0.42	0.09	0.09	1.00	0.73	0.66	0.58	0.35	0.11
	RF	1.00	1.00	1.00	0.60	0.37	0.32	1.00	0.94	0.83	0.79	0.69	0.49
	SIS	1.00	1.00	1.00	0.48	0.08	0.06	0.22	0.08	0.04	0.04	0.03	0.00
	ISIS	1.00	1.00	1.00	0.32	0.02	0.00	0.16	0.06	0.03	0.03	0.02	0.00
	CC-SIS	1.00	1.00	1.00	0.50	0.08	0.06	0.86	0.40	0.31	0.14	0.11	0.02
32	ICC-SIS	1.00	1.00	1.00	0.30	0.05	0.02	0.83	0.27	0.18	0.12	0.08	0.00
	DC-SIS	1.00	1.00	1.00	0.54	0.22	0.16	1.00	0.87	0.73	0.76	0.55	0.28
	RF	1.00	1.00	1.00	0.66	0.47	0.37	1.00	0.97	0.93	0.88	0.80	0.66
	SIS	1.00	1.00	1.00	0.54	0.12	0.08	0.22	0.08	0.04	0.04	0.04	0.00
	ISIS	1.00	1.00	1.00	0.32	0.03	0.00	0.16	0.06	0.04	0.03	0.02	0.00
	CC-SIS	1.00	1.00	1.00	0.54	0.13	0.08	0.86	0.46	0.33	0.20	0.12	0.04
48	ICC-SIS	1.00	1.00	1.00	0.33	0.08	0.02	0.83	0.28	0.19	0.14	0.10	0.01
	DC-SIS	1.00	1.00	1.00	0.60	0.28	0.22	1.00	0.91	0.81	0.81	0.65	0.40
	RF	1.00	1.00	1.00	0.71	0.58	0.41	1.00	0.99	0.94	0.94	0.88	0.77

Summarized from Tables 1, 2, 3, 4, 5 and 6, we conclude that RF performs uniformly best among the six feature screening approaches. In particular, RF stands out under harder conditions. We know that Sim 2 and Sim 4 have more harsh conditions than that of Sim 1 and Sim 3. However, if comparing the left panel and right panel of these tables, we notice that while the majority of approaches get caught by the traps of complexity, RF obtains either similar or even better results.

### Mice HDL GWAS project

Epidemiological studies have consistently shown that the level of plasma high density lipoprotein (HDL) cholesterol is negatively correlated with the risks of coronary artery disease and gallstones [36–38]. Therefore, there has been considerable interest in understanding genetic mechanisms contributing to variations in HDL levels. Zhang et al. published an open resource outbred mouse database with 288 Naval Medical Research Institute (NMRI) mice and 44,428 unique SNP genotypes (available at http://cgd.jax.org/datasets/datasets.shtml) [39]. A total of 581,672 high density SNP were initially genotyped by the Novartis Genomics Factory using the Mouse Diversity Genotyping Array [40]. Quality control was made and only polymorphic SNPs with minor allele frequency greater than 2 %, Hardy-Weinberg equilibrium *χ*^2^<20, and missing values less than 40 % were retained [41]. Moreover, identical SNPs within a 2 Mb interval were collapsed. This left 44,428 unique SNP genotypes for final analysis.

**Fig. 1:**
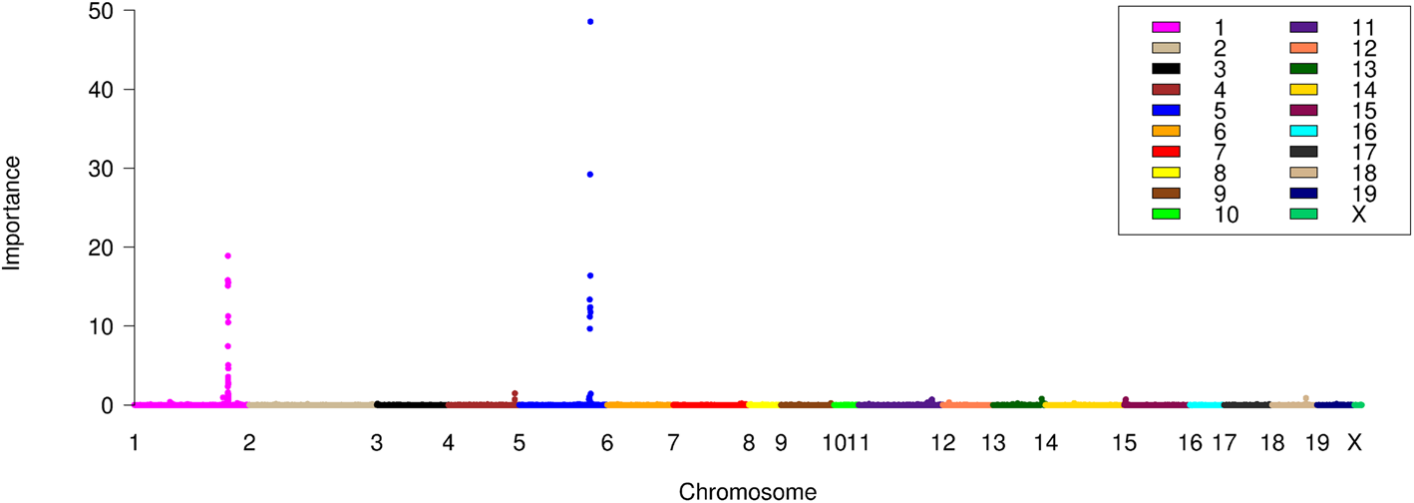
PVIM based Manhattan Plot. Variable importance measure of SNPs obtained from RF for the NMRI mice HDL cholesterol GWA study. Each color corresponds to one chromosome

We implemented RF as the feature screening tool to this data to compare our findings with the highly validated discoveries in current literature. Figure 1 depicts the PVIM for each SNP as a function of the SNP location (in Mb) for 19 chromosomes. The two dramatic peaks detected by RF are located at *Chr1* at *Mb173* and *Chr5* at *Mb125*, which are exactly the same as other reports for the same data, but with a couple of advantages. First, type I error is not a problem here. In traditional *p*-value based Manhattan plots, there exist lots of signals surrounding the peaks and these signals can be so dense and strong (slightly above the threshold line) that it is hard to determine them as type I error or not. However, we notice that the signals in Fig 1 are polar opposites, with only two peaks standing out and all other SNPs shrinking towards zero. With such a clear trend, no one will doubt whether all SNPs other than the two peaks are type I error or truly causative genetic variants. Second, we achieve the same results more directly. Zhang et al. identified three loci as significant, with two loci on Chromosome 1 (Chr 1) and a single locus on Chromosome 5 (Chr 5) (see Fig. 3 of [39]). However, after an extensive comparisons of three analysis, linear trend test, two way ANOVA, and EMMA, they claimed that the significant findings in Mb182 of Chr1 were spurious [39]. Third, we achieve the same results with much less computational speed and burden. Zhang et al. made multiple correction by using a simulation approach [42] as well as the permutation approach [43], both of which are very time consuming by generating thousands of replication samples.

There is one difference in findings worth mentioning here. Zhang et al. had the highest peak achieved at Chr 1 and the second highest peak at Chr 5. We found the opposite. The *p*-values obtained from single-locus models (linear trend test, two way ANOVA, and EMMA) all found that the peak at Chr 1 has smaller *p*-values and hence is more significant than that of Chr 5. However, single-locus models only rank features by their marginal effects without considering interactive, correlative, and polygenic effects. On the contrary, RF gives a rank based on the overall importance, considering the individual effect of each SNP as well as accounting for the mutual joint effects of all other SNPs in a multivariate sense. Confirmed from Tables 1, 2, 3, 4, 5 and 6 of the simulation results, we think that RF ranks the peak of Chr 5 the highest because it is more important in terms of its overall effects (marginal, interactive, correlative, and polygenic effects) for the phenotype.

The two dramatic peaks detected by RF are also highlighted by a *Nature Reviews Genetics* report [44]. Chr5 locus at Mb125, the highest peak in Fig. 1, is located in the same locus as QTL *Hdlq1* found by Su et al. and Korstanje et al. [45, 46]. In addition, they conclude that *Scarb1*, the well known gene involved in HDL metabolism, is the causal gene underlying *Hdlq1* by haplotype analysis, gene sequencing, expression studies, and a spontaneous mutation [47, 48]. Chr1 locus at Mb173, the second highest peak in Fig. 1, is the major determinant of HDL, which has been detected as QTL *Hdlq15* in inbred mouse strains multiple times. Numerous mouse crosses have linked HDL to this region, and *Apoa2* has been identified as the gene underlying this QTL [37, 38, 45].

The Manhattan plot using − log10(*p*) as the rule to test significance of each SNP has been widely used in almost all current GWAS literature [16, 44, 49–52]. Instead, we make Manhattan plot from PVIM as an alternative rule to judge significance. A possible argument may come from the threshold or cutoff level used to determine the significance. If using *p*-value, the traditional determination is to judge if − log10(*p*) passes the threshold of −*log*_10_(0.05/*p*). However, the threshold is quite controversial in RF area. There is no a clear solution for it yet. Chen et al. combined the PVIM with permutation to compute the *p*-values so that the threshold can be available [13]. However, they did not support it using solid theoretical derivations and simulation verifications.

Although the threshold of PVIM of RF is not feasible, it does not affect us to use PVIM based Manhattan plot to draw importance conclusions given the following concerns. 1) The threshold determination is not the key interest of the feature screening approach. Like aforementioned five popular feature screening approaches, a pre-specified number of candidates is picked and there is no requirement of close parameter estimating or significance determining in feature screening. 2) Jiang et al. compared RF with the *p*-values got from B statistic and reported an extremely strong consistency between the *p*-value and the importance measure. They claimed that larger importance corresponds to smaller *p*-value of B statistic [11, 33]. It indicated that the importance of RF can give an alternative significance measure of association between SNPs and phenotype. 3) Lunetta et al. found that RF outperforms Fisher’s Exact test when interactive effects exist, in terms of power and type I error [32]. It again illustrated the comparable performance of PVIM with a *p*-value approach. 4) The threshold of *p*-value approach is obtained by multiple correction, which may not be reliable for a ultra-high dimensional number of SNPs. For example, Bonferroni correction was claimed to be too conservative for large number of tests. The PVIM avoids the multiple correction issue. 5) After having a closer investigation on the Fig 1, we notice that the difference between significance vs non-signifiance is very obvious. Therefore, it is not necessary to use thresholds to determine significance versus non-significance. The two polarized separate is not an accidental because RF tends to have small type I error without losing power.

## Conclusion

In this article, we investigated the performance of a forest-based feature screening approach for detecting epistatic, correlative, and polygenic effects for large-scale genome data. Besides the difficulties caused by high dimension, the challenges of epistasis are tripled when hundreds of thousands of SNPs are genotyped. The most popular single-locus models are lack of power, mainly because they ignore the complex mutual effects among SNPs. Extensive studies have already been performed to handle epistasis, such as Brute-force search, exhaustive search, greedy search, MDR, CPM, and so on. However they mainly target for manageable number of features and will lose power for ultrahigh dimension of features. Marchini et al. proposed to exhaustively search all possible 2-way interactive combinations [2]. We agree that this exhaustive search is able to detect all important 2-way interactions. However, it cannot track higher order interactions or more complex structures. Additionally, the search load will be astronomical if the dimension is ultrahigh.

Due to its high efficiency, easy implementation, and great accuracy, feature screening has received much attention for reducing the number of features from huge to moderate through importance rankings [26]. However, majority current feature screening approaches rank the features by univariate measure and neglect the features with weak marginal but complex overall effects. By controlling the difficulty levels through four different monte carlo simulation studies, we compared RF with five other popular feature screening approaches. To make the comparisons consistent, we used the same criteria, same simulation design, and same simulated data for all six approaches. We conclude that the forest-based feature screening performs nicely when nonlinear, interactive, correlative, and other complex associations of response and features exist. In addition, we noticed that the advantages of RF are more manifested when the data conditions are more harsh. We also examined a real mice HDL whole genome data and further confirmed the advantages of RF compared to other current studies for the same data. The human data can be easily extended.

## Methods

The purpose of feature screening is to recognize a small set of features that are truly associated with response from a big pool with ultrahigh dimension. By individually defining a surrogate measure for underlying association between response and each feature, feature screening ranks features from the most important to the least important.

### Sure independence screening (SIS)

SIS ranks features based on componentwise regression or correlation learning. Each feature is used independently to decide how useful it is for predicting the response variable. Let *w*=(*w*_1_,…,*w*_*p*_)^*T*^=*X*^*T*^*y* be a vector that is obtained by component wise regression, where *X* is the standardized feature matrix. Then, *w* is the measure of marginal correlations of features with the response. The features are sorted based on the componentwise magnitude of the absolute value of *w* in a decreasing order [26].

### Iterative sure independence screening (ISIS)

Fan and Lv pointed out the drawbacks of the SIS: an important feature marginally uncorrelated but jointly correlated with the response can not be picked by SIS. The spurious features not directly associate with the response but in high correlation with a causative feature will likely be selected by SIS [26]. The iterative SIS (ISIS) was proposed to address these drawbacks. The idea of ISIS is to iterate the SIS procedure conditional on previously selected features. To be more specific, first select a small subset *k*_1_ of features, then regress the response over these features. Treat the residuals as the new response and apply the same method to the remaining *p*−*k*_1_ features to pick another small subset *k*_2_ of features. Keep on the iteration until the union of all steps achieve the prespecified size [26].

### Conditional correlation sure independence screening (CC-SIS)

Consider how the case effect of response on a feature is related with a covariate, i.e. the parameter *β* can be a function of certain important covariate *u*. Now the conditional correlation between the response and each feature is defined as

$$\rho(x_{j},y|u)=\frac{cov(x_{j},y|u)}{\sqrt{cov(x_{j},x_{j}|u)~cov(y,y|u)}},~j=1,\ldots,p. $$

Define the marginal measure as *w*=(*w*_1_,…,*w*_*p*_)^*T*^=*E*{*ρ*^2^(*x*_*j*_,*y*|*u*)} and rank the importance of features based on the estimated value of *w* in a decreasing order [30].

### Iterative conditional correlation sure independence screening (ICC-SIS)

Since CC-SIS is based on the top of SIS, it also exists similar drawbacks of the SIS. In order to select the marginally uncorrelated but jointly correlated features and also reduce the effect of collinearity, ICC-SIS was proposed. The idea of ICC-SIS is exactly same as ISIS, but performs CC-SIS during each iteration of residual fitting [30].

### Distance correlation sure independence screening (DC-SIS)

The dependence strength between two random vectors can be measured by the distance correlation (Dcorr) [29]. Szekely et al. showed that the Dcorr of two random vectors equals zero if and only if these two random vectors are independent. The distance covariance is defined as

$$dcov^{2}(y,x_{j})=\int ||\phi_{y,x_{j}}(t,s)-\phi_{y}(t)\phi_{x_{j}}(s)||^{2}~w(t,s)dtds, $$

where *ϕ*_*y*_(*t*) and $\phi _{x_{j}}(s)$ are the respective characteristic functions of *y* and *x*_*j*_, and $\phi _{y,x_{j}}(t,s)$ is the joint characteristic function of (*y*,*x*_*j*_), and

$$w(t,s)=\left\{{c_{1}^{2}}~||t||^{2} ~||s||^{2}\right\}^{-1}, $$

with *c*_1_=*π*, and ||·|| stands for the Euclidean norm. Then the Dcorr is defined as

$$dcorr(y,x_{j})=\frac{dcov(y,x_{j})}{\sqrt{dcov(y,y)~dcov(x_{j},x_{j})}}. $$

DC-SIS approach does not assume any parametric model structure and works well for both linear and nonlinear associations. In addition, it works well for both categorical and continuous data without making assumptions about the data type.

### Random forest (RF)

RF has been widely used for modeling complex joint and interactive associations between response and multiple features [12, 32, 33, 53]. In particular, many nice properties of RF make it an extremely attractive tool for genome studies: the data structure of response and features can be a mixture of categorical and continuous variables; it can nonparametrically incorporate complex nonlinear associations between feature and response; it can implicitly incorporate joint and unknown complex interactions among a large number of features (higher orders or any structure); it is able to handle big data with a large number of features but limited sample size; it can implicitly accommodate highly correlated features; it is less prone to over-fitting; it has good predictive performance even when the majority of features are noise; it is invariant to monotone transformations of the features; it is robust to changes in its tuning parameters; it performs internal estimation of error, so does not need to assess classification performance by cross-validation, and hence greatly reduces computational time [13, 32, 53, 54].

Using an ensemble method (also called committee method), RF creates multiple classification and regression trees (CARTs). The detailed process of RF can be described in the following steps: Step 1, a bootstrap sample of size *n* is randomly drawn with replacement from the original data. The remaining non-selected sample or “Out-of-Bag" sample (OOB) is about 30 *%* on average. Step 2, a classification tree is grown on the bootstrap sample without trimming, by recursively splitting data into distinct subsets with one parent node branched into two child nodes. At each node, a fixed number of features is randomly chosen without replacement from all original features, with “mtry" pre-specifying how many features are chosen. The best split is based on minimizing the mean square prediction error. Step 3, previous two steps are repeated to grow a pre-specified number of trees and make a decision based on the majority vote of all trees (classification) or average results over all trees (regression). Step 4, the prediction accuracy is computed using OOB samples [53].

As an output of the RF, the permutation PVIM, considering the difference in prediction accuracy before and after permuting the jth (*j*=1,…,*p*) feature *X*_*j*_ is defined as

$$PVIM_{t}(X_{j})=\frac{\sum_{i\in B_{t}}\left(Y_{i}-\hat{Y}_{ti}\right)^{2}-\sum_{i\in B_{t}}\left(Y_{i}-\hat{Y}^{*}_{ti}\right)^{2}}{|B_{t}|}. $$

Here *B*_*t*_ is the OOB sample for tree *t*, *t*=1,…,*ntree*. $\hat {Y}_{\textit {ti}}$ is the predicted class for observation *i* got from tree *t* before permuting *X*_*j*_ and $\hat {Y}^{*}_{\textit {ti}}$ is the predicted class after permuting *X*_*j*_. The final importance measure is averaged over all trees

$$PVIM(X_{j})=\sum_{t=1}^{ntree} PVIM_{t}(X_{j})/ntree. $$

If one feature is randomly permuted, its original association with the response will be broken. Therefore, the idea of PVIM is this: if one feature is an important factor for response, the prediction accuracy should decrease substantially when using its permuted version and all other non-permuted features to predict the OOB sample.

According to the asymptotic theory of RF, RF is sparse when sample size approaches to infinity with a fixed number of features *p* (i.e. only a small number of causal features is truly associated with the response) [55], which matches the goal of feature screening. The PVIM gives an important measure for each feature, based on their level of associations with response, and hence can be used for feature screening [56]. The PVIM assess each variable’s overall impacts by counting not only marginal effects, but also all other complex correlative, interactive, and joint effects, without requiring model structures or explicitly putting interactive terms into the model [32]. The overall effects of each feature are assessed implicitly by the multiple features in the same tree and also by the permuting process when all other features are left unchanged but kept in the same model. Therefore, the variable with weak marginal but strong overall effects will be assigned a high PVIM value [31, 32].

## Availability of supporting data

The data set that we analyzed was freely download from http://cgd.jax.org/datasets/datasets.html) [39].

## Abbreviations

GWAS: Genome-wide association studies
RF: Random forest
PVIM: Permutation variable importance measure (PVIM)
SNPs: Single nucleotide polymorphisms
MDR: Multifactor-dimensionality reduction
CPM: Combinatorial partitioning method
SIS: Sure independence screening
ISIS: Iterated sure independence screening
DC-SIS: Distance correlation sure independence screening
CC-SIS: Conditional correlation sure independence screening
ICC-SIS: CC-SIS: Iterated conditional correlation sure independence screening
HDL: High density lipoprotein
NMRI: Naval Medical Research Institute
Chr: Chromosome
Dcorr: Distance correlation
OOB: “Out-of-Bag" sample
CART: Classification and regression trees
